# A boy and his mother with lipoprotein glomerulopathy: Two case reports and literature review

**DOI:** 10.1097/MD.0000000000041628

**Published:** 2025-02-21

**Authors:** Shuya Zhang, Lulu Fan, Ziwei Li, Tiantian Liu, Xiaoping Jing, Qingyin Guo

**Affiliations:** a The Pediatric Hospital, The First Affiliated Hospital of Henan University of Chinese Medicine, Zhengzhou, China; b The Pediatric Medical College, Henan University of Chinese Medicine, Zhengzhou, China; c Department of Traditional Chinese Medicine, Shanghai Children’s Hospital, Shanghai Jiao Tong University, School of Medicine, Shanghai, China.

**Keywords:** apolipoprotein E, case report, lipoprotein glomerulopathy, literature review

## Abstract

**Rationale:**

Lipoprotein glomerulopathy (LPG) is a rare genetic kidney disorder. Here, we report a boy and his mother with LPG.

**Patient concerns:**

A 6-year-old boy was admitted to our hospital with a history of 6 months of experiencing foamy urine without apparent cause.

**Diagnoses:**

Urinalysis revealed 3+ protein and 2+ occult blood. A 24-hour urinary protein quantification measured 1110 mg. Other laboratory tests revealed that the level of serum albumin was 43.6 g/L, triglycerides 4.31 mmol/L were elevated, and high-density lipoprotein cholesterol 0.71 mmol/L were reduced, whereas total cholesterol and low-density lipoprotein cholesterol levels were normal. Renal biopsy revealed glomerular capillary loop expansion with lipoprotein thrombi on light microscopy, variable-sized vacuoles within the capillary loops on electron microscopy, positive Oil Red O staining, and positive immunofluorescence staining for ApoE. The mother of the patient had a history of uremia 5 years ago. Genetic testing confirmed a deletion of 9 nucleotides (CAAGCTGCG) in exon 4 of the ApoE gene at positions c.480–488 of the boy and his mother, resulting in a deletion of 3 amino acids (Lys143–Arg145del) in the ApoE amino acid sequence at positions 143–145, which was same variant as ApoE Tokyo/Maebashi.

**Interventions:**

The boy showed significant improvement after treatment with fenofibrate and telmisartan, with urine protein turning negative after 1 week and blood lipid levels returning to normal after 4 weeks.

**Outcomes:**

During 1 year follow-up period, the results of urine routine examination and blood lipid profile remained within normal ranges.

**Lessons:**

LPG is a rare and easily misdiagnosed kidney disease with no clinical characteristics. Early diagnosis by kidney biopsy and whole gene test is conducive to early detection and diagnosis, reducing missed diagnosis and misdiagnosis, and improving the long-term prognosis of patients.

## 1. Introduction

Lipoprotein glomerulopathy (LPG), is a rare autosomal dominant inherited kidney disease with incomplete penetrance. It is more commonly observed in China and Japan.^[1]^ In 1989, Saito et al^[2]^ first reported 2 cases of LPG, characterized by significant dilation of glomerular capillary lumens with lipoprotein thrombi and abnormal blood lipid levels in patients. Subsequently, it was discovered that LPG is caused by mutations in the apolipoprotein E (ApoE) gene.^[3]^ To date, approximately 14 different ApoE gene variants have been identified in association with LPG.^[4,5]^ Patients with LPG often exhibit abnormalities in lipid metabolism (similar to type III hyperlipoproteinemia) and elevated concentrations of ApoE, as well as varying degrees of proteinuria. The main pathological feature is the presence of a large amount of lipoprotein thrombi in the glomerulus.^[6]^ At present, the main treatment in clinical practice focuses on symptomatic therapy such as reducing blood lipids, decreasing proteinuria, and delaying the progression of renal function. Recently, our hospital treated a boy with LPG and discovered a mutation site in the ApoE gene, which was inherited from his mother who had uremia. This mutation resulted in the deletion of 3 amino acids (Lys143-Arg145del) of ApoE, which was same variant as ApoE Tokyo/Maebashi. This article also discusses the clinical and pathological characteristics as well as the treatment of this condition, and reviews the more comprehensive clinical data on LPG from both domestic and international literature. It summarizes the clinical features, diagnosis, treatment, and prognosis of LPG.

## 2. Case reports

A 6-year-old boy was admitted to our hospital in April 2023 after 6 months of experiencing foamy urine without apparent cause. Urinalysis revealed 3+ protein and 2+ occult blood. A 24 hours urinary protein quantification measured 1110 mg. Other laboratory tests revealed that level of serum albumin was 43.6 g/L, triglycerides (TG) 4.31 mmol/L were elevated, and high-density lipoprotein cholesterol (HDL-C) 0.71 mmol/L were reduced, whereas normal total cholesterol and low-density lipoprotein cholesterol (LDL-C) levels were normal. Laboratory tests during admission showed decreased hemoglobin (96 g/L), 2+ proteinuria and 3+ occult blood in the urine, elevated 24-hour urinary protein (1429.2 mg), slightly decreased serum albumin (30.7 g/L), elevated total cholesterol and triglycerides (6.54 mmol/L and 2.76 mmol/L respectively), and elevated LDL-C (4.45 mmol/L). Color Doppler ultrasonography revealed enlarged kidneys bilaterally (left, 106 × 51 × 49 mm; right, 104 × 56 × 52 mm), with mild diffuse enhancement of parenchymal echogenicity. The patient’s mother was diagnosed with uremia before 5 years and has been undergoing hemodialysis. No history of renal insufficiency in other family members was reported.

**Figure 1.**
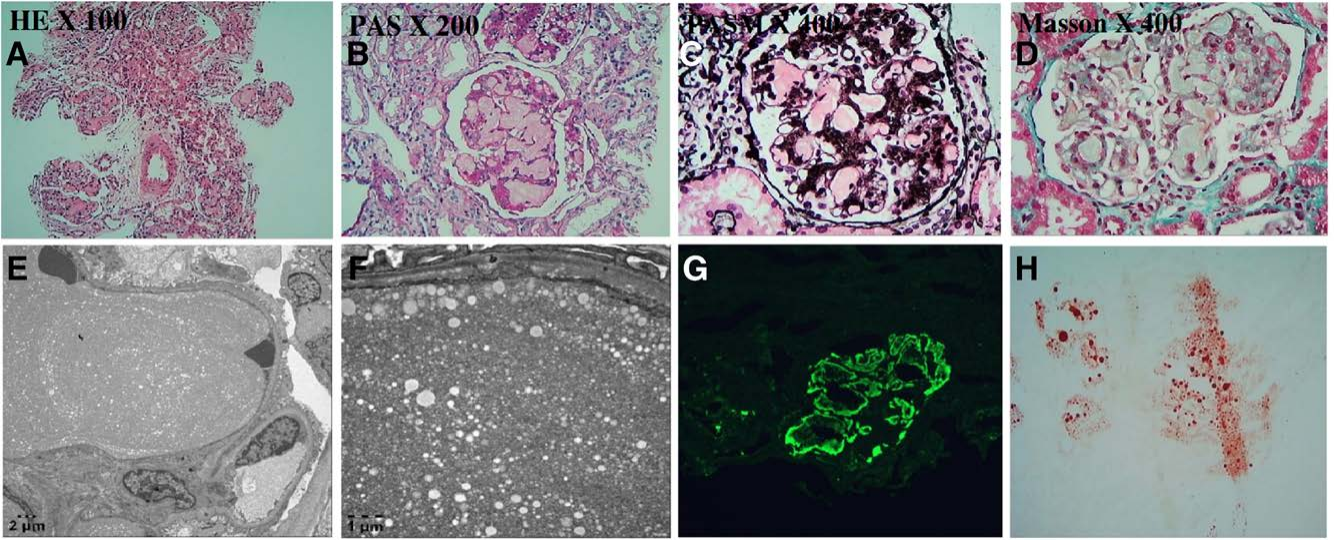
Renal biopsy in the boy with LPG. (A–D) Light microscopy. The renal biopsy tissue submitted for examination was stained with HE, PAS, PASM, and Masson stains. Enlargement of the glomerular volume and marked expansion of capillary loops were observed. The lumina were filled with lightly stained, vacuolated thrombus-like material. There was also proliferation of mesangial cells and matrix, with focal mesangial dissolution. Under the electron microscope, the endothelial cells of the capillaries display evident vacuolar degeneration. The capillary loops are open, partially dilated, and contain a large amount of lipoprotein vacuoles. (E, F) Electron microscope. (G) ApoE: luminal substance positivity. (H) Oil red O staining: positive staining of luminal contents. HE = hematoxylin and eosin, LPG = lipoprotein glomerulopathy, PAS = periodic acid-schiff, PASM = periodic acid-silver methenamine.

Under light microscopy, 17 glomeruli from a renal biopsy revealed no evidence of glomerulosclerosis or segmental sclerosis; however, they showed increased volume, extensive capillary dilation, and the presence of lightly stained, vacuolated thrombus-like material within the lumens. Mesangial cell and matrix proliferation, with focal mesangial dissolution, were evident. The epithelial cells in the vessel walls showed no significant proliferation, and no crescent formation was evident (Fig. 1A–D). Immunofluorescence staining for immunoglobulins M, G, and A, and complement proteins C3, C4, and C1q was negative. Electron microscopy revealed significant vacuolar degeneration of the glomerular endothelial cells, open and partially dilated capillary loops, and abundant proteinaceous material in the lumens in the form of lipid-like vacuoles were evident (Fig. 1E, F). The luminal material stained positive for ApoE (Fig. 1G) and Oil Red O (Fig. 1H). Taken together, these results were consistent with the diagnosis of LPG. Treatment with Tripterygium wilfordii glycosides, telmisartan, and piperazine ferulate resulted in no improvements in blood lipids and urinary protein.

**Figure 2.**
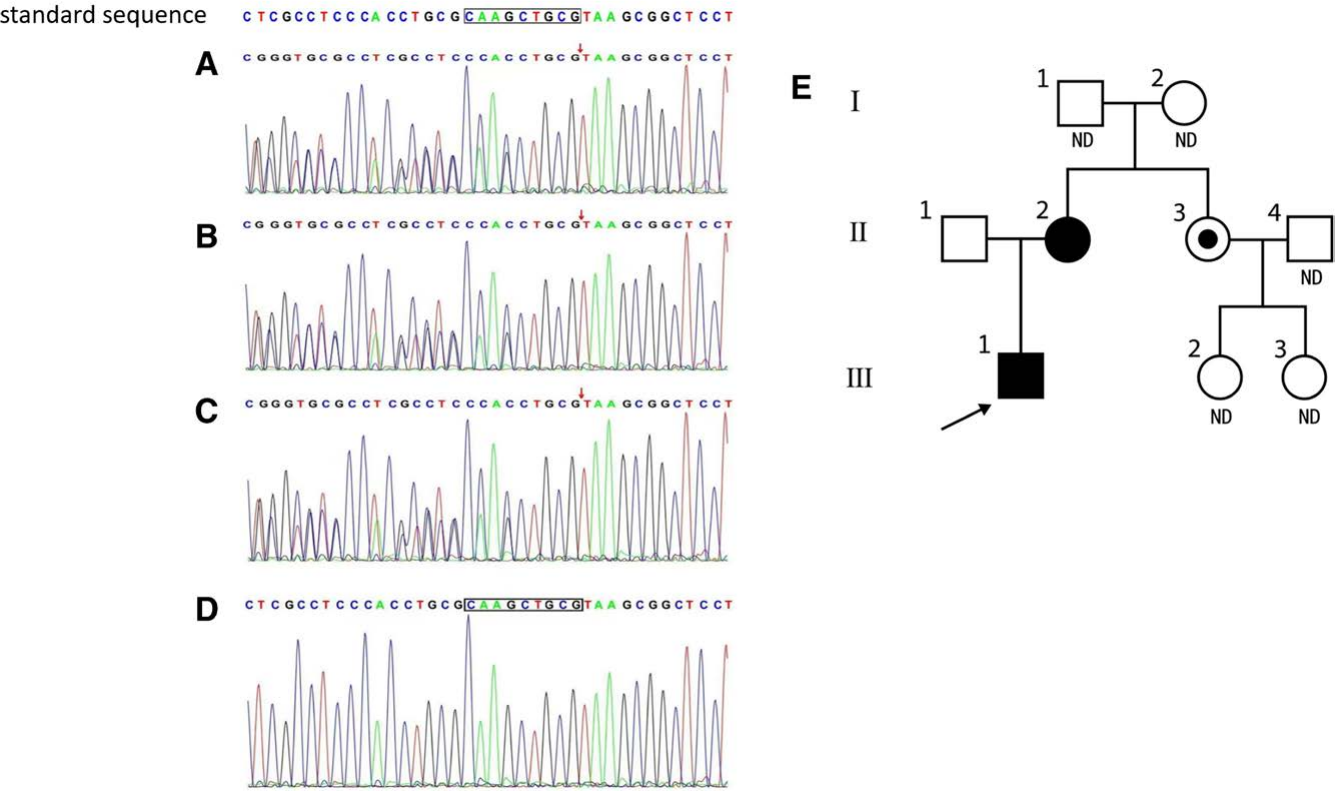
DNA sequence analysis of ApoE gene mutation and the pedigree chart of the child. The standard sequence displays the normal ApoE gene sequence. Genetic testing confirmed the presence of a mutation in the ApoE gene in the child, with a deletion of 9 nucleotides (CAAGCTGCG) in exon 4 (A), resulting in the deletion of 3 amino acids (Lys143-Arg145del) in ApoE. The mother (B) and aunt (C) of the child have the same ApoE gene mutation, while his father does not have the mutation and is wild-type (D). Genetic screening was performed on the child, his parents, and his aunt. III-1 is the proband (indicated by an arrow). Squares and circles represent males and females, respectively. Empty symbols represent unaffected family members, while black symbols represent heterozygous patients with ApoE (Lys143-Arg145del) mutation. The dot symbol indicates asymptomatic carriers of this mutation. The proband and his mother and aunt are heterozygous for the ApoE gene. The father is healthy and has a wild-type ApoE gene. The proband does not have any siblings, and his grandparents refused genetic testing. II-4, III-2, and III-3 did not undergo genetic testing due to economic reasons and have no history of kidney disease. Since the lack of testing, they have been marked as ND (undetermined; E). DNA = deoxyribonucleic acid, ND = undetermined.

Gene testing of peripheral blood from family members revealed a mutation in the ApoE gene of the proband: c.480 (exon4) to c.488 (exon4) delCAAGCTGCG (p.Lys161-Arg163del, Lys143-Arg145del). This mutation was identical to the previously reported APOE Tokyo/Maebashi (141-143del/142-144del). The proband’s mother and aunt, but not the father, carried the same ApoE gene mutation. However, the aunt’s routine blood, urine, and liver and kidney function tests showed no abnormalities. The proband’s father, grandparents, aunt, and aunt’s daughters have not exhibited symptoms of LPG to date (Fig. 2).

The patient’s treatment plan was subsequently adjusted to include once-daily oral fenofibrate (200 mg) and twice-daily telmisartan (40 mg per dose). One week later, upon follow-up, the urinary protein and occult blood results were found to be negative. Additional laboratory results included 24-hour urinary protein, 158.98 mg; serum albumin, 31.4 g/L; total cholesterol, 6.26 mmol/L (increased); LDL-C, 4.32 mmol/L (increased); HDL-C, 1.43 mmol/L; and TG level of 1.46 mmol/L. One month later, urinary protein and occult blood remained negative on routine follow-up testing, with these additional results: 24-hour urine protein 30 mg, and serum albumin 44.6 g/L. Blood lipids were within their normal ranges: TG, 1.43 mmol/L; total cholesterol, 4.09 mmol/L; LDL-C, 1.65 mmol/L; and HDL-C, 1.34 mmol/L. At 1 year of follow-up, routine urinary testing, 24-hour urinary protein, and blood lipids were normal.

## 3. Discussion and conclusion

LPG is an extremely rare genetic kidney disease characterized by abnormal deposition of lipoprotein in the glomeruli, resulting in the obstruction of the capillaries being the main histopathological feature. Approximately 270 cases of LPG have been reported to date worldwide. The age of onset varies widely, ranging from 4 to 72 years, with approximately equal sex distribution and familial clustering.^[1,3]^ The main characteristics of LPG include proteinuria, hematuria, or both, elevated blood lipids, and increased serum ApoE.^[7]^ Without early intervention, renal failure can be progressive.^[8]^ The pathogenic mechanisms of LPG have not been fully elucidated; however, genetic variations in the ApoE gene is considered to be the main hypothesis.

The ApoE glycoprotein is composed of 299 amino acids, encoded by the ApoE gene on chromosome 19. It is primarily synthesized in the liver, but can also be produced by mesangial cells in the kidney.^[9]^ There are 3 common isoforms of ApoE, namely E2, E3, and E4, with their synthesis controlled by the ε2, ε3, and ε4 alleles, respectively. The type of isoform is determined by 2 amino acid residues at positions 112 and 158 on the fourth exon of the ApoE gene. ApoE3 is the most common subtype, with Arg and Cys at positions 112 and 158, respectively. ApoE2 has Cys at both positions, while ApoE4 has Arg at both positions.^[10]^

**Figure 3.**
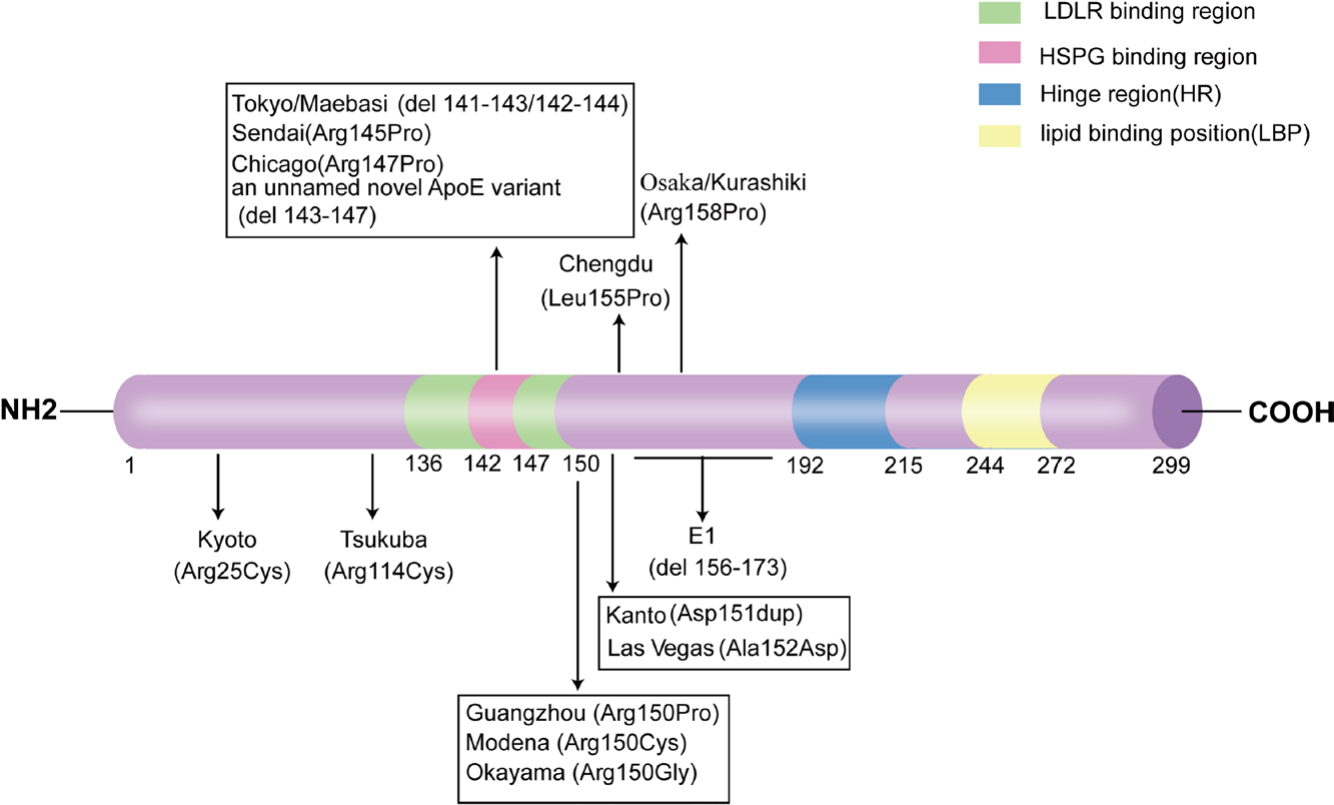
The structure of ApoE and gene mutations in different regions are described. 14 different ApoE mutations are reported. The green region represents the low-density lipoprotein receptor (LDLR) binding region (amino acids 136-150), the red region represents the heparan sulfate proteoglycan (HSPG) binding region (amino acids 142-147), the blue region represents the hinge region (HR; amino acids 192-215), the yellow region represents the lipid binding position (LBP; mino acids 244-272). Arg (arginine), Cys (cysteine), Pro (proline), Gly (glycine), Leu (leucine), Ala (alanine), Asp (aspartic acid), del (deletion), dup (duplication). Ala = alanine, Arg = arginine, Asp = aspartic acid, Cys = cysteine, del = deletion, dup = duplication, Gly = glycine, HR = hinge region, HSPG = heparan sulfate proteoglycan, LBP = lipid binding position, LDLR = low-density lipoprotein receptor, Leu = leucine, Pro = proline.

ApoE can be divided into 2 structural domains, each with distinct structures and functions, involved in lipid transport and metabolism (Fig. 3). The N-terminal domain contains the low-density lipoprotein receptor (LDLR) binding region and the heparan sulfate proteoglycan (HSPG) binding region. The C-terminal domain is closely associated with lipid binding, with the lipid binding position binding to triglycerides and cholesterol, together forming chylomicrons, very low-density lipoproteins (VLDL), intermediate-density lipoproteins, and some high-density lipoproteins.^[11]^ Triglyceride-rich lipoproteins (TRLs) in plasma include chylomicrons secreted by the intestine and VLDL secreted by the liver, mainly responsible for transporting triglycerides to adipose tissue for storage and serving as an energy source for skeletal and cardiac muscles. After entering the bloodstream, TRLs are broken down by lipoprotein lipase (LPL), removing a large amount of triglycerides, phospholipids, and ApoC, and taking up cholesterol esters and ApoE to form smaller and denser remnants of lipoproteins. ApoE on remnants of lipoproteins mediates the uptake of TRL by liver cells through the HSPG pathway or binding to LDLR in liver cells, thereby reducing circulating lipid levels.^[12,13]^ ApoE3 and ApoE4 have normal binding ability to LDLR, while ApoE2 exhibits a binding defect with LDLR, leading to impaired lipid clearance and its association with type III hyperlipoproteinemia.^[14]^

Currently, multiple ApoE gene mutations have been identified in LPG patients. The first reported mutation is ApoE Sendai (Arg145Cys), which is a missense mutation. This mutation replaces the G at position 145 of the DNA sequence with C, resulting in the substitution of arginine with cysteine at position 145 of ApoE.^[15]^ It is considered a major mutation in LPG. Another major mutation is ApoE Kyoto (Arg25Cys), which changes the arginine at position 25 of ApoE to cysteine. Although this site is not located in the binding region between ApoE and LDLR, the cysteine at this site can form disulfide bridges with cysteines at other sites, disrupting the tertiary structure of ApoE.^[16]^ ApoE Sendai and ApoE Kyoto are the 2 most common mutations, and in terms of receptor binding ability, they exhibit only 5% of the activity of normal proteins when binding to LDLR, leading to the aggregation and clearance impairment of lipoproteins.^[1]^

So far, there have been 14 reported APOE gene variations associated with the onset of LPG worldwide, including missense mutations and in-frame deletions. Most of the mutations are located at the LDLR binding site and its surrounding region, particularly the HSPG binding domain, such as ApoE Tokyo (del 141-143),^[17]^ ApoE Maebashi (del 142-144),^[18]^ ApoE Sendai (Arg145Pro),^[15]^ ApoE Chicago (Arg147Cys),^[19]^ ApoE Guangzhou (Arg150Pro),^[20]^ ApoE Modena (Arg150Cys),^[21]^ ApoE Okayama (Arg150Gly),^[22]^ and an unnamed novel ApoE variant (del 143-147).^[5]^ These mutations may potentially result in decreased binding capacity between ApoE and LDLR. The other mutations are located outside the LDLR binding region, such as ApoE Kyoto (Arg25Cys),^[16]^ ApoE Tsukuba (Arg114Cys),^[23]^ ApoE Kanto (Asp151dup),^[24]^ ApoE Las Vegas (Ala152Asp),^[25]^ ApoE Chengdu (Leu155Pro),^[26]^ ApoE1 (del 156-173)^[27]^ and ApoE Osaka/Kurashiki (Arg158Pro).^[28]^ These mutations may potentially alter the spatial conformation and stability of the ApoE protein (Fig. 3). The substitution of proline with other amino acids is an important mutation, such as ApoE Sendai, ApoE Chicago, ApoE Chengdu, ApoE Guangzhou, and ApoE Osaka/Kurashiki, as it may lead to a decrease in α-helical content, exposure of hydrophobic surfaces, thermodynamic instability, and aggregation of ApoE, resulting in LPG.^[29]^ Currently, only 1 Chinese study reported no apparent ApoE gene mutations in 17 LPG patients, while other patients exhibited ApoE gene mutations,^[30]^ indicating that other factors may also induce LPG.

Patients with LPG often present with nephrotic syndrome, with varying degrees of microscopic hematuria and type III hyperlipoproteinemia, but only affecting the kidneys without systemic changes in hyperlipidemia. In addition to significant elevations in plasma triglyceride and cholesterol levels, there is also a marked increase in ApoE, and the therapeutic effects of glucocorticoids or immunosuppressants are unsatisfactory. The unique pathological manifestations of the kidneys and genetic testing play an important role in the diagnosis of lipoprotein glomerulopathy. The typical features of LPG include glomerular capillary loop dilation with layered lipoprotein thrombi and the rarity of foam cells derived from macrophages. The lipoprotein thrombi can form a fingerprint-like appearance under blood flow impact. Immunofluorescence staining shows positive ApoE and/or ApoB in the mesangial area and within the capillary loops of the glomerulus.

Currently, treatment for LPG aims to reduce proteinuria and lipid levels, delaying the progression of renal dysfunction. Glucocorticoids, immunosuppressants, and anticoagulants have all demonstrated poor efficacy.^[3,31]^ Previous case reports that found combined treatment with glucocorticoids and immunosuppressants ineffective used intensified treatment with lipid-lowering drugs (including fibrates) to provide clinical and histological relief for patients. In those cases, serum cholesterol, triglycerides, and ApoE decreased, and consecutive renal biopsies demonstrated complete disappearance of lipoprotein thrombi.^[22,32–34]^ Fibrates drugs include fenofibrate, bezafibrate, etc, which lower serum triglyceride levels and increase HDL-C levels by activating peroxisome proliferator-activated receptor alpha and upregulating LPL expression, and improve proteinuria. Compared with the control group, the fibrates treatment group has significantly higher survival rate and kidney survival rate during the 3-year follow-up period.^[35]^ A study in China demonstrated the feasibility of staphylococcal protein A immunoadsorption therapy,^[36]^ because staphylococcal protein A can bind to the Fc of IgG, thereby clearing circulating ApoE and deposited ApoE in renal tissues,^[37]^ and regular treatment can significantly delay the progression of renal disease. Another report indicates that plasma exchange using heparin-induced extracorporeal lipoprotein precipitation system can effectively treat LPG, significantly reducing cholesterol and triglycerides. This is because the heparin-induced extracorporeal lipoprotein precipitation system can activate LPL and hepatic triglyceride lipase through heparin, making TRL (such as VLDL and intermediate-density lipoproteins) easier to clear.^[21]^ However, immune adsorption and plasma exchange are currently not widely used in clinical practice due to the difficulties in operation, high cost, and risk of infection. Literature reports that there have been a total of 6 cases of LPG patients undergoing kidney transplantation worldwide, with 5 cases experiencing LPG recurrence in the transplanted kidney, and ApoE abnormalities in the recipients may induce lipoprotein thrombosis in the transplanted kidney.^[8]^

Our patient had a relatively short disease course, presenting clinically with nephrotic syndrome accompanied by anemia. Following treatment with fenofibrate combined with telmisartan, proteinuria quickly turned negative and blood lipid levels returned to normal. No other signs of extrarenal tissue lipid deposition in our patient were observed, although his anemia suggests a possible association with lipid deposition in the bone marrow, affecting hematopoiesis. In contrast, the patient’s mother had rapidly progressed to end-stage renal disease after a renal injury. Although the boy’s aunt carries a variation in the ApoE gene, she has not developed LPG or experienced renal dysfunction, indicating that other factors can also induce LPG.

LPG is a rare and poor-prognosis kidney disease. Initial diagnosis often leads to misdiagnosis as primary nephrotic syndrome, and caution should be exercised for patients who do not respond well to glucocorticoids and immunosuppressive therapy. Renal biopsy is the gold standard for diagnosing LPG, whose main pathologic manifestations are glomerular capillary loop expansion and the presence of layered lipoprotein thrombi. Currently, Lipid-lowering therapy, immunoadsorption, plasma exchange, and even kidney transplantation provide only symptomatic improvement and a delay of disease progression, and the long-term efficacy of these treatments remains unclear. ApoE plays a critical role in lipoprotein metabolism, and LPG is strongly associated with ApoE gene mutations. Therefore, effective treatment for LPG must target the genetic level, which is also the focus of our future research. In-depth study is needed to explore more effective treatment methods.

## Author contributions

**Writing – original draft:** Shuya Zhang.

**Data curation:** Ziwei Li, Tiantian Liu.

**Supervision:** Lulu Fan.

**Writing – review & editing:** Xiaoping Jing, Qingyin Guo.

## References

[1] Li MS Li Y Liu Y Zhou XJ Zhang H. An updated review and meta analysis of lipoprotein glomerulopathy. Front Med (Lausanne). 2022;9:905007. 35602473 10.3389/fmed.2022.905007 PMC9120586

[2] Saito T Sato H Kudo K. Lipoprotein glomerulopathy: glomerular lipoprotein thrombi in a patient with hyperlipoproteinemia. Am J Kidney Dis. 1989;13:148–53. 2644825 10.1016/s0272-6386(89)80134-9

[3] Saito T Matsunaga A Oikawa S. Impact of lipoprotein glomerulopathy on the relationship between lipids and renal diseases. Am J Kidney Dis. 2006;47:199–211. 16431249 10.1053/j.ajkd.2005.10.017

[4] Wang R Zhao C Chen W Liu Z Xie F. A novel apolipoprotein E mutation, ApoE Ganzhou (Arg43Cys), in a Chinese son and his father with lipoprotein glomerulopathy: two case reports. J Med Case Rep. 2022;16:78. 35193676 10.1186/s13256-022-03302-0 PMC8864814

[5] Xie W Xie Y Lin Z Xu X Zhang Y. A novel apolipoprotein E mutation caused by a five amino acid deletion in a Chinese family with lipoprotein glomerulopathy: a case report. Diagn Pathol. 2019;14:41. 31092271 10.1186/s13000-019-0820-6 PMC6521367

[6] Matsunaga A Saito T. Apolipoprotein E mutations: a comparison between lipoprotein glomerulopathy and type III hyperlipoproteinemia. Clin Exp Nephrol. 2014;18:220–4. 24570178 10.1007/s10157-013-0918-1

[7] Tsimihodimos V Elisaf M. Lipoprotein glomerulopathy. Curr Opin Lipidol. 2011;22:262–9. 21464714 10.1097/MOL.0b013e328345ebb0

[8] Saito T Matsunaga A Fukunaga M Nagahama K Hara S Muso E. Apolipoprotein E-related glomerular disorders. Kidney Int. 2020;97:279–88. 31874799 10.1016/j.kint.2019.10.031

[9] Chen G Paka L Kako Y Singhal P Duan W Pillarisetti S. A protective role for kidney apolipoprotein E. Regulation of mesangial cell proliferation and matrix expansion. J Biol Chem. 2001;276:49142–7. 11579084 10.1074/jbc.M104879200

[10] Jia L Xu H Chen S. The APOE ε4 exerts differential effects on familial and other subtypes of Alzheimer’s disease. Alzheimers Dement. 2020;16:1613–23. 32881347 10.1002/alz.12153 PMC7984370

[11] Mahley RW Innerarity TL Rall SC Jr Weisgraber KH. Plasma lipoproteins: apolipoprotein structure and function. J Lipid Res. 1984;25:1277–94. 6099394

[12] Illingworth DR. Lipoprotein metabolism. Am J Kidney Dis. 1993;22:90–7. 8322800 10.1016/s0272-6386(12)70173-7

[13] Feingold KR. Lipid and lipoprotein metabolism. Endocrinol Metab Clin North Am. 2022;51:437–58. 35963623 10.1016/j.ecl.2022.02.008

[14] Nguyen D Dhanasekaran P Nickel M. Molecular basis for the differences in lipid and lipoprotein binding properties of human apolipoproteins E3 and E4. Biochemistry. 2010;49:10881–9. 21114327 10.1021/bi1017655 PMC3025481

[15] Oikawa S Matsunaga A Saito T. Apolipoprotein E Sendai (arginine 145-->proline): a new variant associated with lipoprotein glomerulopathy. J Am Soc Nephrol. 1997;8:820–3. 9176854 10.1681/ASN.V85820

[16] Matsunaga A Sasaki J Komatsu T. A novel apolipoprotein E mutation, E2 (Arg25Cys), in lipoprotein glomerulopathy. Kidney Int. 1999;56:421–7. 10432380 10.1046/j.1523-1755.1999.00572.x

[17] Konishi K Saruta T Kuramochi S. Association of a novel 3-amino acid deletion mutation of apolipoprotein E (Apo E Tokyo) with lipoprotein glomerulopathy. Nephron. 1999;83:214–8. 10529627 10.1159/000045513

[18] Ogawa T Maruyama K Hattori H. A new variant of apolipoprotein E (apo E Maebashi) in lipoprotein glomerulopathy. Pediatr Nephrol. 2000;14:149–51. 10684367 10.1007/s004670050032

[19] Sam R Wu H Yue L. Lipoprotein glomerulopathy: a new apolipoprotein E mutation with enhanced glomerular binding. Am J Kidney Dis. 2006;47:539–48. 16490634 10.1053/j.ajkd.2005.12.031

[20] Luo B Huang F Liu Q. Identification of apolipoprotein E Guangzhou (arginine 150 proline), a new variant associated with lipoprotein glomerulopathy. Am J Nephrol. 2008;28:347–53. 18046082 10.1159/000111828 PMC2785906

[21] Russi G Furci L Leonelli M. Lipoprotein glomerulopathy treated with LDL-apheresis (Heparin-induced Extracorporeal Lipoprotein Precipitation system): a case report. J Med Case Rep. 2009;3:9311. 20062740 10.1186/1752-1947-3-9311 PMC2803834

[22] Kinomura M Sugiyama H Saito T. A novel variant apolipoprotein E Okayama in a patient with lipoprotein glomerulopathy. Nephrol Dial Transplant. 2008;23:751–6. 18045818 10.1093/ndt/gfm675

[23] Hagiwara M Yamagata K Matsunaga T. A novel apolipoprotein E mutation, ApoE Tsukuba (Arg 114 Cys), in lipoprotein glomerulopathy. Nephrol Dial Transplant. 2008;23:381–4. 17967799 10.1093/ndt/gfm735

[24] Yokochi A Matsunaga A Kanemoto K Tominaga N Uda S Saito T. Lipoprotein glomerulopathy with a novel apolipoprotein E variant, APOE Kanto (Asp 151dup). CEN Case Rep. 2024. doi: 10.1007/s13730-024-00920-z. 10.1007/s13730-024-00920-z PMC11958863 39141311

[25] Bomback AS Song H D’Agati VD. A new apolipoprotein E mutation, apoE Las Vegas, in a European-American with lipoprotein glomerulopathy. Nephrol Dial Transplant. 2010;25:3442–6. 20624773 10.1093/ndt/gfq389

[26] Wu H Yang Y Hu Z. The novel apolipoprotein E mutation ApoE Chengdu (c.518T>C, p.L173P) in a Chinese patient with lipoprotein glomerulopathy. J Atheroscler Thromb. 2018;25:733–40. 10.5551/jat.41996 PMC6099066 29398675

[27] Ando M Sasaki J Hua H. A novel 18-amino acid deletion in apolipoprotein E associated with lipoprotein glomerulopathy. Kidney Int. 1999;56:1317–23. 10504484 10.1046/j.1523-1755.1999.00677.x

[28] Mitani A Ishigami M Watase K Minakata T Yamamura T. A novel apolipoprotein E mutation, ApoE Osaka (Arg158 Pro), in a dyslipidemic patient with lipoprotein glomerulopathy. J Atheroscler Thromb. 2011;18:531–5. 21325775 10.5551/jat.7377

[29] Georgiadou D Stamatakis K Efthimiadou EK. Thermodynamic and structural destabilization of apoE3 by hereditary mutations associated with the development of lipoprotein glomerulopathy. J Lipid Res. 2013;54:164–76. 23110818 10.1194/jlr.M030965 PMC3520522

[30] Chen S Liu ZH Zheng JM Zhang X Li LS. A complete genomic analysis of the apolipoprotein E gene in Chinese patients with lipoprotein glomerulopathy. J Nephrol. 2007;20:568–75. 17918142

[31] Li W Wang Y Han Z Luo C Zhang C Xiong J. Apolipoprotein e mutation and double filtration plasmapheresis therapy on a new Chinese patient with lipoprotein glomerulopathy. Kidney Blood Press Res. 2014;39:330–9. 25300642 10.1159/000355810

[32] Ieiri N Hotta O Taguma Y. Resolution of typical lipoprotein glomerulopathy by intensive lipid-lowering therapy. Am J Kidney Dis. 2003;41:244–9. 12500244 10.1053/ajkd.2003.50016

[33] Arai T Yamashita S Yamane M. Disappearance of intraglomerular lipoprotein thrombi and marked improvement of nephrotic syndrome by bezafibrate treatment in a patient with lipoprotein glomerulopathy. Atherosclerosis. 2003;169:293–9. 12921981 10.1016/s0021-9150(03)00194-1

[34] Matsunaga A Furuyama M Hashimoto T Toyoda K Ogino D Hayasaka K. Improvement of nephrotic syndrome by intensive lipid-lowering therapy in a patient with lipoprotein glomerulopathy. Clin Exp Nephrol. 2009;13:659–62. 19603250 10.1007/s10157-009-0207-1

[35] Hu Z Huang S Wu Y. Hereditary features, treatment, and prognosis of the lipoprotein glomerulopathy in patients with the APOE Kyoto mutation. Kidney Int. 2014;85:416–24. 24025644 10.1038/ki.2013.335

[36] Xin Z Zhihong L Shijun L. Successful treatment of patients with lipoprotein glomerulopathy by protein A immunoadsorption: a pilot study. Nephrol Dial Transplant. 2009;24:864–9. 18840890 10.1093/ndt/gfn555

[37] Atkins KL Burman JD Chamberlain ES. S. aureus IgG-binding proteins SpA and Sbi: host specificity and mechanisms of immune complex formation. Mol Immunol. 2008;45:1600–11. 18061675 10.1016/j.molimm.2007.10.021

